# Two Sides of the Same Coin: TFIIH Complexes in Transcription and DNA Repair

**DOI:** 10.1100/tsw.2010.46

**Published:** 2010-04-13

**Authors:** Alexander Zhovmer, Valentyn Oksenych, Frédéric Coin

**Affiliations:** IGBMC Department of Functional Genomics, CNRS/INSERM/ULP, BP 163, 67404 Illkirch Cedex, C. U. Strasbourg, France

**Keywords:** TFIIH, helicase, DNA repair, genomic instability

## Abstract

TFIIH is organized into a seven-subunit core associated with a three-subunit Cdk-activating kinase (CAK) module. TFIIH has roles in both transcription initiation and DNA repair. During the last 15 years, several studies have been conducted to identify the composition of the TFIIH complex involved in DNA repair. Recently, a new technique combining chromatin immunoprecipitation and western blotting resolved the hidden nature of the TFIIH complex participating in DNA repair. Following the recruitment of TFIIH to the damaged site, the CAK module is released from the core TFIIH, and the core subsequently associates with DNA repair factors. The release of the CAK is specifically driven by the recruitment of the DNA repair factor XPA and is required to promote the incision/excision of the damaged DNA. Once the DNA lesions have been repaired, the CAK module returns to the core TFIIH on the chromatin, together with the release of the repair factors. These data highlight the dynamic composition of a fundamental cellular factor that adapts its subunit composition to the cell needs.

## INTRODUCTION

Our genome is vulnerable to an array of DNA-damaging agents that affect fundamental cellular processes, such as DNA replication and transcription. To counteract the deleterious effects of these agents, cells are armed with several DNA repair pathways that protect us from cancer and accelerated aging[1]. Each of these DNA repair pathways removes structure-specific DNA lesions. The nucleotide excision repair (NER) pathway removes bulky adducts, including cisplatin lesions and 6-4 photoproducts generated by UV light, through two related subpathways[2]. The general global genome repair (GGR) removes DNA damages from the entire genome, while the transcription-coupled repair (TCR) corrects lesions located on actively transcribed genes[3]. The importance of DNA repair mechanisms in genome stability is emphasized by the existence of several repair-deficient disorders. Deficiency in NER results in three rare genetic diseases: xeroderma pigmentosum (XP), trichothiodystrophy (TTD), and Cockayne syndrome (CS)[4]. XP patients are highly photosensitive and display a 1000-fold increased risk of developing skin cancers[4]. TTD patients are mildly photosensitive, but present neurological disorders and sulfur-deficient brittle hair and nails caused by the reduced level of cysteine-rich matrix proteins[5]. CS patients are also mildly photosensitive, but harbor neurological problems, growth failure, and premature aging[6]. XP and TTD patients are deficient both in GGR and TCR (with the exception of XP-C patients who are only deficient in GGR, see below). Pure CS patients are deficient only in TCR. GGR is then fully functional in persons with this syndrome, but they die prematurely from progeria. This leads to the hypothesis that the low level of residual lesions that block transcription in CS cells promotes apoptosis. This premature cell death would protect CS patients from cancer at the expense of aging.

In GGR, XPC-HR23B detects the damage-induced DNA distortion in the genome, followed by the opening of the DNA by the XPB and XPD ATPases/helicases of the transcription/repair factor TFIIH[7]. XPA and RPA are then recruited to the repair complex and assist in the expansion of the DNA bubble around the damage[8,9]. Next, the endonucleases XPG and XPF generate cuts in the 3′ and 5′ sides of the lesion, respectively[10,11], thereby causing the removal of a 27-nts(±2)-long damaged oligonucleotide[12,13]. Finally, the resynthesis machinery fills the DNA gap[14]. In TCR, blockage of transcribing RNA polymerase II (RNPII) on the damaged DNA template is thought to initiate the repair reaction in a process that requires, in addition to TFIIH, XPA, XPG and XPF, the TCR-specific proteins CSB and CSA[2]. The transcription-coupled NER pathway is independent from the XPC-HR23B complex, thereby explaining the strict GGR defect harbored by the XP-C patients. Among the NER factors, TFIIH has attracted the most interest, owing to its additional roles in several fundamental cellular processes.

## TFIIH: A MULTISUBUNIT FACTOR WITH SEVERAL CELLULAR TASKS

TFIIH is a ten-subunit complex[15,16], essential for the transcription of RNAI- and II-dependent genes and for the NER pathway[17,18]. The TFIIH complex can be divided into two subcomplexes: the core and the CAK. The core TFIIH includes XPB, p62, p52, p44, p34, and the repair-specific TTDA subunit. XPD links the core to the Cdk-activating kinase (CAK) module composed of Cdk7 (cyclin-dependent kinase), cyclin H, and MAT1 (ménage à trois) (Fig. 1). Mutations in three TFIIH subunits give rise to XP, TTD, or CS; mutations in XPB are associated with a combined XP-CS phenotype or with TTD; mutations in XPD are associated with XP, XP-CS, or TTD; and mutations in TTDA are associated only with TTD[4].

In RNPII transcription, TFIIH joins the preinitiation complex composed of RNPII and the general transcription factors TFIIA, TFIIB, TFIID, TFIIE, and TFIIF (Fig. 1). There, TFIIH is involved in several processes ranging from initiation, promoter escape, and early elongation stages[19], to transcription reinitiation[20] and formation of gene loops[21]. Besides its roles in RNPII transcription, TFIIH is also involved in the transcription of ribosomal genes by RNPI. The reconstituted *in vitro* RNPI transcription system requires TFIIH[18] and the complex localizes in the nucleolus at sites of active ribosomal gene transcription[22]. TFIIH was also reported to be part of a complex containing RNPI and the NER factors CSB and XPG[23], but the precise role of TFIIH during RNPI transcription yet remains unknown. In NER, TFIIH belongs to the dual incision complex composed of XPC-HR23B, XPA, RPA, XPG, and ERCC1-XPF, and is involved in the opening of the DNA around the damage (Fig. 1).

Four TFIIH subunits harbor enzymatic activities required for transcription and DNA repair (Fig. 1). Cdk7 phosphorylates RNPII and certain nuclear receptors, and thereby regulates basal and activated transcription[24,25]. XPB and XPD are ATPases/helicases involved in DNA opening[7]. The ATPase activity of XPB is required for anchoring TFIIH to the damaged DNA[26,27], while the helicase activity of XPD opens DNA around the lesion[28]. The enzymatic activity of XPD is not required for RNPII transcription and this subunit is therefore believed merely to act as a structural component of the TFIIH complex and to regulate the kinase activity of Cdk7[29,30]. Finally, p44 was described as an E3 ubiquitin ligase in yeast[31]. This activity is suggested to be important for the survival of cells after exposure to UV light or methyl methanesulfonate (MMS).

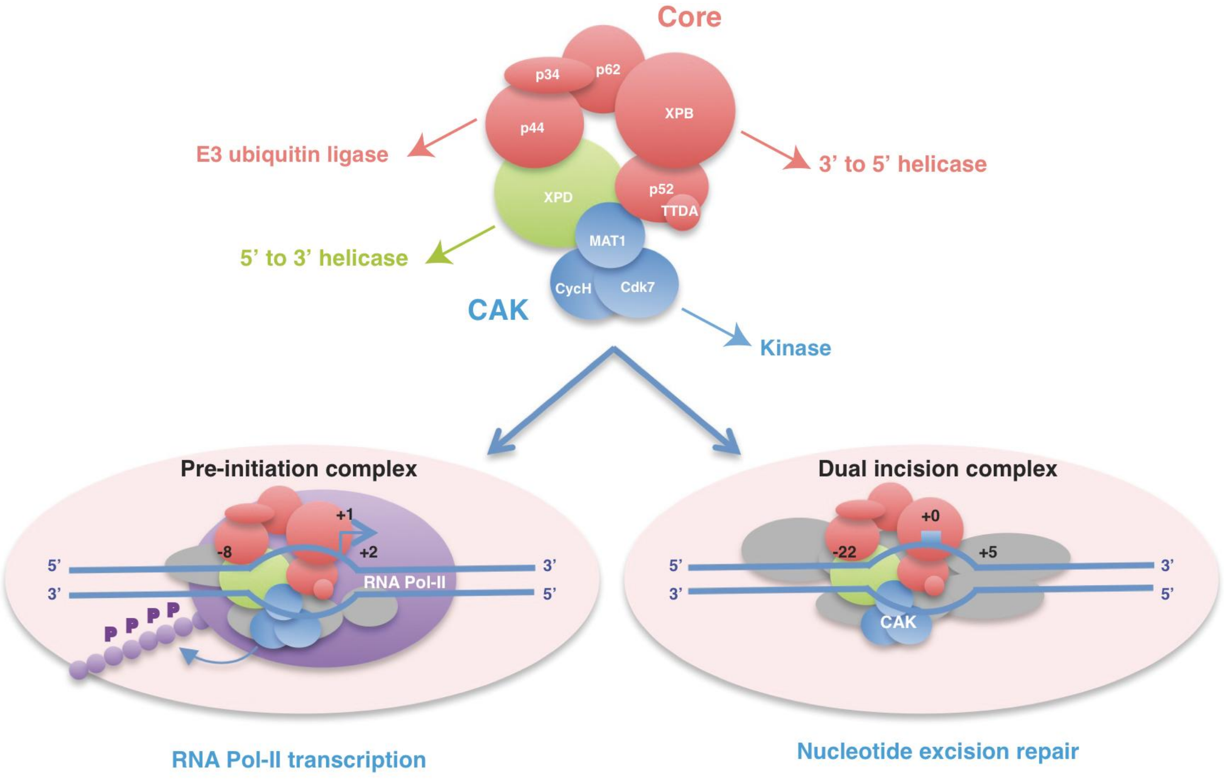

**FIGURE 1.** A multisubunit and tasks complex. TFIIH is a ten-subunit complex composed of a core (in red; XPB, p62, p52, p44, p34, and TTDA) associated to the CAK (in blue; Cdk7, CycH, and MAT1) through MAT1 and the XPD subunit (in green). Four enzymatic activities are found in TFIIH: XPB and XPD are 3' to 5' and 5' to 3' helicases, Cdk7 is a kinase, and p44 has been described as an E3 ubiquitin ligase in yeast. The complex is involved both in RNPI- and II-dependant transcription and in nucleotide excision repair. The role of TFIIH in RNPI transcription in unknown. In RNPII-dependant transcription, TFIIH opens DNA around the promoter in the preinitiation complex (–8 to +2 relatively to the transcription start site +1) and phosphorylates the carboxyl terminal domain of the RNPII (purple) to license transcription. In NER, TFIIH opens DNA around the lesion (–22 to +5 relatively to the lesion in blue square) and assists ERCC1-XPF for the 5' incision[77].

The enzymatic activities of TFIIH are highly regulated either by members of the complex or by DNA repair factors (Table 1). The p52 subunit of TFIIH stimulates the ATPase activity of XPB[28]. TTDA is a NER-specific factor that is recruited to the TFIIH complex after a genotoxic attack[32,33]. During NER, TTDA stimulates the ATPase activity of XPB through a direct interaction with p52[34]. The TTDA-interacting domain of p52 also binds DNA, and the addition of TTDA triggers dissociation of p52 from DNA. We hypothesize that the recruitment of TTDA to TFIIH provokes a conformational change of the complex that leads to a *bona fide* interaction between p52 and XPB, and to the stimulation of its ATPase activity[35]. The repair factor XPC also regulates the ATPase activity of XPB[36]. On the other hand, p44 stimulates XPD helicase activity, required to unwind DNA around the damage[37]. The MAT1 protein of the CAK module has been shown to inhibit XPD helicase and this negative effect might be compensated by p44 stimulation[38]. The importance of these regulations is pointed out by the existence of mutations in XP patients that impair the XPB-p52[28] or XPD-p44[37,39] interactions, leading to cancers and premature aging.

## THE CAK COMPLEX: THE DOUBLE LIFE OF A KINASE

The CAK complex is composed of Cdk7, cyclin H, and MAT1[40] associated with U1 snRNA[41]. Cdk7 can be found in three different complexes: in CAK alone (50% of Cdk7), in CAK together with core TFIIH (40% of Cdk7), or in CAK with XPD alone (10% of Cdk7) (Fig. 2). Several factors, including

**TABLE 1**
**TFIIH Enzymatic Activities, Their Functions, and Their Regulators**

| Subunit | Activity | Function | Partners | Regulators |
|---|---|---|---|---|
| XPB | DNA-dependent ATPase | Opening of the DNA around the promoter; anchoring of TFIIH to damaged DNA | p52, p62* | p52 (stimulates the ATPase)[28], TTDA (stimulates the ATPase in a TFIIH context)[33], XPC (stimulates the ATPase on DNA)[36] |
| XPD | 3' to 5' helicase | Opening of DNA around the damage | p44, p52, MAT1 | p44 (stimulates the helicase)[37,39], MAT1 (inhibits the helicase)[38] |
| p44 | E3 ubiquitin ligase** | Transcription answer after damage | XPD, p34 | p34 (stimulates the E3 ubiquitin ligase)[31]** |
| Cdk7 | Kinase | Basal transcription; activated transcription | Cyclin H, MAT1 | Cyclin H (stimulates the kinase activity)[52], TFIIE(stimulates the kinase)[52], MAT1 (stabilizes the association between Cdk7 and cyclin H)[52] |

* Unpublished data.

** In yeast.

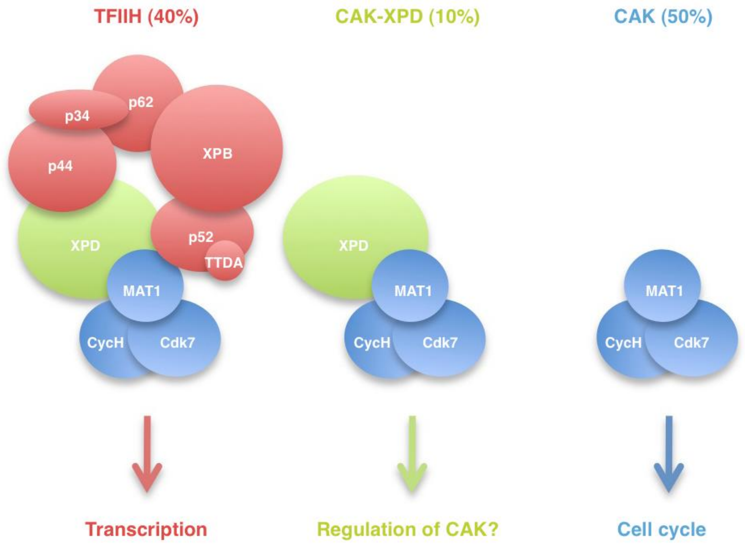

**FIGURE 2.** The three Cdk7-containing complexes. The Cdk7 kinase can be found in three different complexes, TFIIH, CAK-XPD, and CAK, that represent 40, 10, and 50% of the Cdk7-containing complexes (personal data), respectively. When Cdk7 is in TFIIH, it functions in transcription, while it functions in cell cycle progression when it is in CAK alone or associated with XPD.

cyclin H, MAT1, TFIIE, the Mediator[42], XPD[43], and U1 snRNA[44], regulate the activity of Cdk7. Phosphorylation of Cdk7 at threonine 170 is required for the CAK activity[45], while phosphorylation of serine 164 by Cdk1 and Cdk2 inhibits Cdk7 activity in cell cycle progression[46]. MAT1 interacts with both Cdk7 and cyclin H, and thereby stabilizes the assembly of the CAK complex[47,48].

Surprisingly, free CAK and CAK interacting with core TFIIH have significantly different substrate specificity. Free CAK acts as a Cdk-activating kinase and phosphorylates Cdk1, Cdk2, Cdk4, and Cdk6 involved in cell cycle progression[49,50,51,52]. When it interacts with the core, CAK preferentially phosphorylates the carboxy-terminal domain (CTD) of the RNPII large subunit rpb1[53,54]. The CTD consists of multiple repeats of the conserved sequence YSPTSPS, which attract both mRNA- and histone-modifying enzymes, depending on their phosphorylation state[55]. Whereas the serine 5 of the CTD is phosphorylated by TFIIH during transcription initiation, Rtr1 phosphatase removes it during elongation[56]. Recently, another Cdk7-dependent phosphorylation was demonstrated on serine 7 of the CTD in both yeast and mammalian cells[57,58,59]. As a component of TFIIH, CAK also phosphorylates nuclear receptors, including the retinoic acid receptors $\alpha$ and $\gamma$, the estrogen receptor $\alpha$[25,60,61], the peroxysome proliferator-activated receptor[62], and the thyroid receptor[63], thereby supporting the transcription of the nuclear receptor–dependent genes. Cdk7 also activates the vitamin D receptor indirectly by phosphorylating the Ets1 coactivator[64]. The role of the CAK-XPD complex is more elusive, but it seems to represent an inactive version of the CAK complex. Indeed, evidence points to a negative regulation of CAK activity by XPD. By this means, XPD would regulate the progression of the cell cycle at the mitotic phase[65].

While the function of CAK in transcription is well documented, its role in NER is contradictory. Microinjection of anti-Cdk7 immunoglobulins into human fibroblasts affects both transcription and NER[66], but dual incision assay can be reconstituted without the CAK complex. These data suggest that the TFIIH core is sufficient to perform excision of the damaged DNA *in vitro*[67]. Indeed, when Cdk7 expression is knocked down using siRNA, fully functional repair of UV lesions is maintained, whereas transcription activation of UV-inducible genes is significantly inhibited[68]. Also, the kinase activity of CAK is detrimental to the dual incision assay efficiency[67], but UV irradiation affects this activity[48]. In yeast, two TFIIH complexes can be purified in undamaged cells: a core TFIIH associated with repair factors that is active in NER and a CAK-associated TFIIH complex that is active in transcription[69]. Do these two TFIIH complexes coexist in mammalian cells? Is the CAK module physically engaged in NER? How does a single TFIIH complex face the task of participating in both transcription and NER?

## TFIIH IN TRANSCRIPTION AND REPAIR: DR. JEKYLL AND MR. HYDE

To understand how the ten subunits that constitute the TFIIH complex participate in repair and/or transcription processes, we utilized an original approach combining chromatin immunoprecipitation and western blotting (ChIP-Western)[70]. With this technique, we were able to analyze the composition of TFIIH on the chromatin before or after UV irradiation. Surprisingly, we observed a dynamic dissociation/reassociation of the CAK complex onto the core TFIIH that correlates with the recruitment/release of the repair factors during the DNA repair reaction[68] (Fig. 3). In the absence of formaldehyde cross-linking, the dissociation of CAK is still observed in the soluble cell fractions, but the UV dose required to dissociate CAK from the core under this condition is much higher than the dose needed to detect the dissociation by ChIP-Western. Also, while repair factors accumulate on the core TFIIH by ChIP, no accumulation was observed on TFIIH in the soluble fraction after UV. These observations advantageously reconcile the yeast and human models by showing that in humans, the free core TFIIH is transient and exists only in the chromatin, during the short period of DNA repair, while in yeast, it exists in the soluble fraction and in absence of DNA damage. In both cases, it is the core TFIIH that participates in DNA repair, in the absence of CAK.

Fifteen minutes after 20J/m2 UV-C irradiation, CAK bound to the TFIIH core reaches a minimum level, whereas the recruitment of repair factors to the TFIIH core reaches its maximum level. At that specific time point, about 70% of TFIIH is involved in DNA repair and does not contain CAK. This early event, together with the fact that the release of CAK is impaired in XP-C cells, in which TFIIH is not recruited to the damaged DNA[71], suggests that the change in TFIIH subunit composition is due to its participation in the removal of the 6-4PP lesions that are eliminated in the first hours after UV irradiation[72]. After the

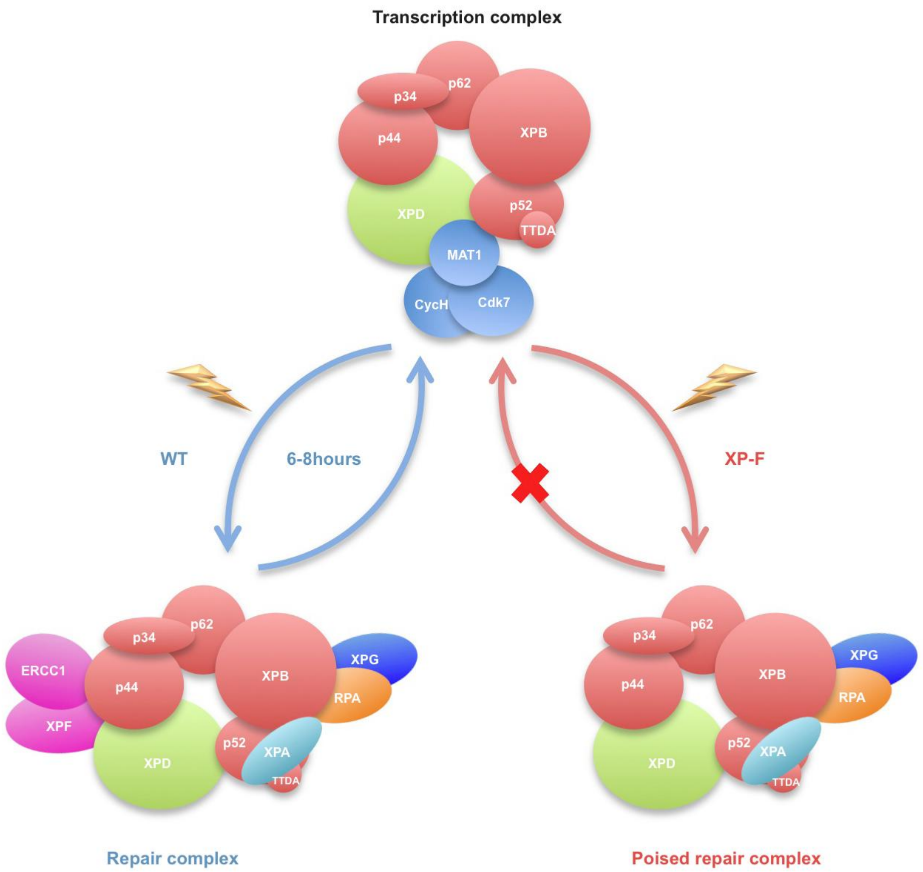

**FIGURE 3.** The dynamic composition of the TFIIH complex. When TFIIH functions in transcription, it contains ten subunits, including the CAK module. When it functions in repair, it does not contain the CAK module, but associates with various DNA repair factors, including XPA, RPA, XPG, and ERCC1-XPF. In a wild-type cell, the transcription complexes are present without DNA damages. The repair complexes appear following the generation of damages on the DNA and persist until the lesions are removed (6–8 h). Mutations in NER proteins, as found in XP patients (here XP-F), cause an accumulation of intermediate poised repair complexes that can persist in the chromatin.

removal of the 6-4PP lesions (6–8 h postirradiation), we observed the dissociation of the repair factors from the core and the return of the CAK. We noticed that mutations in NER proteins, as found in XP patients, cause an accumulation of intermediate repair complexes that can persist for several hours on the chromatin (Fig. 3). The persistence of these intermediate poised complexes may potentially cause prolonged transcription and replication arrests, and their impact on cell survival should be investigated.

The question remains on how and when the CAK is released from the core TFIIH. We reconstituted the dissociation of the CAK from the core TFIIH *in vitro* and provided evidence that the transformation is catalyzed by the presence of XPA, in an ATP-dependent manner. XPA is known as a scaffold protein without enzymatic activity that nevertheless shows preferential association to damaged DNA and is indispensable for DNA incision[73,74]. Our *in vitro* system demonstrated that the release of CAK by XPA is required to trigger the dual incision of the damaged DNA. The recruitment of XPA and the dissociation of CAK may then constitute a major checkpoint in NER that will elicit the removal of lesions (Fig. 4). Consistent with this model, CAK inhibits the helicase activity of XPD[38]. In addition, the recruitment of XPA to the XPC/TFIIH intermediate preincision complex promotes the opening of the damaged DNA[75]. In light of our latest discoveries about CAK release during DNA repair, we propose that detachment of CAK from the core by the damage verification factor XPA will stimulate the helicase/ATPase activities of TFIIH that will lead to opened DNA structures, thereby facilitating DNA repair.

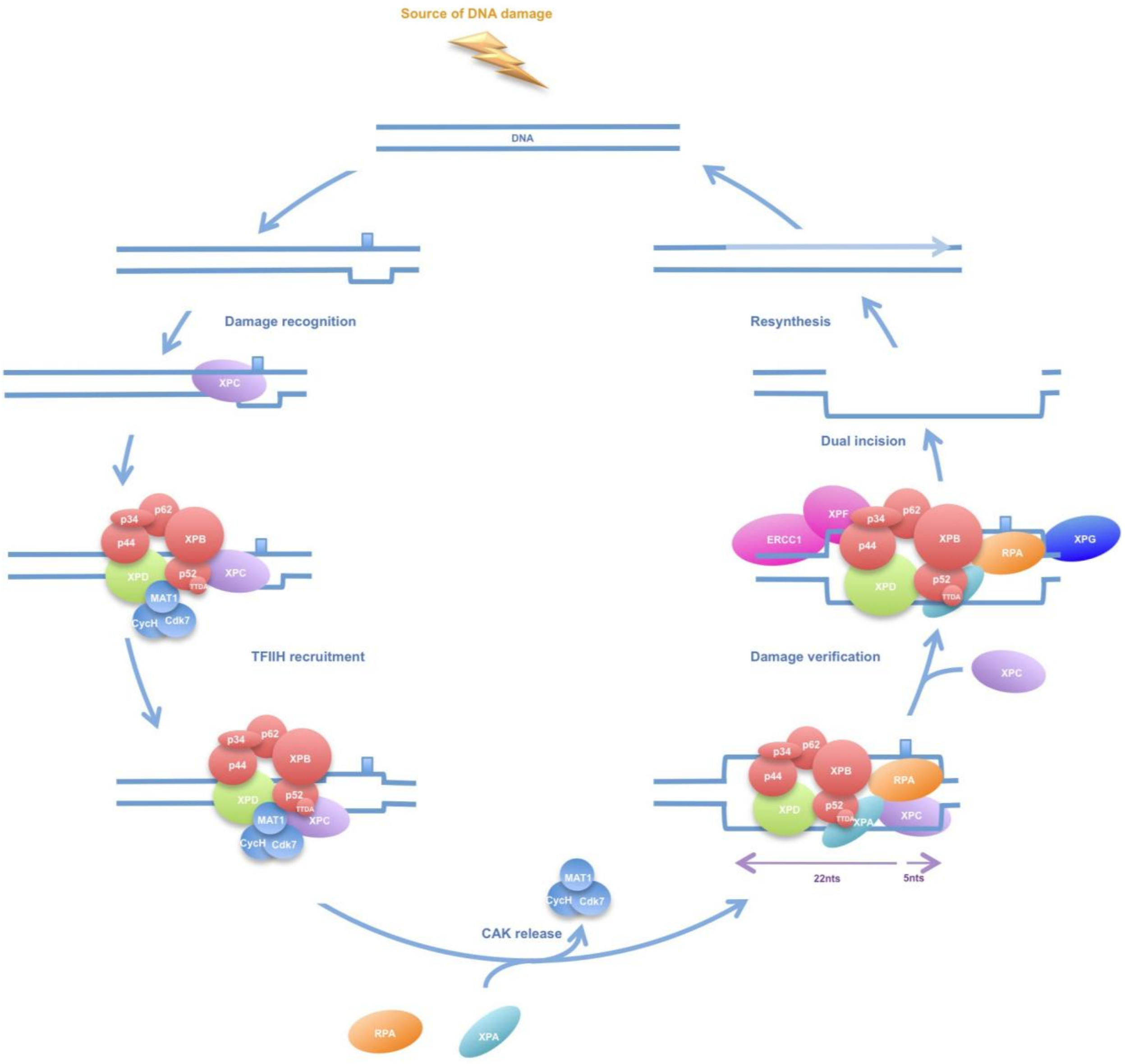

**FIGURE 4.** The nucleotide excision repair pathway. Following exposure to genotoxic agents (e.g., sunlight), a lesion (blue square) is created on the DNA. Then, the damage recognition factor XPC-HR23B interacts with the damaged DNA structure on the opposite strand of the lesion. TFIIH joins XPC-HR23B on the damaged DNA. In the presence of ATP, XPB, and XPD, helicases in TFIIH are involved in the opening of the DNA, allowing the stable association of XPA and RPA, which help to enlarge the opened structure and drive the dissociation of the CAK complex from TFIIH. This dissociation is a prerequisite for the enlargement of the DNA opening that favors the arrival of XPG, mediating the release of XPC-HR23B. The recruitment of XPF-ERCC1 triggers dual incision and excision of the protein-free damaged oligonucleotide. The resynthesis machinery fills the gap and seals the DNA extremities.

## CONCLUSION

Altogether, our results dispel a broadly accepted idea that large nuclear complexes are stable and do not experience large-scale alterations in composition when switching between different cellular processes or different cellular conditions[22]. The flexibility of the TFIIH complex makes it able to participate in various distinct cellular processes. Based on our discovery, we propose that the core TFIIH is involved in the various functions of the complex. The association of the core with different modules enables its engagement in different functions, such as transcription or repair. The two modules, CAK for transcription and TTDA for repair, confer to the core TFIIH and to its helicases the capacity to open unrelated DNA structures, such as promoters or damaged DNA, respectively (Fig. 5). The CAK and the TTDA modules seem to coexist in the same TFIIH complex, but how one negatively influences the activity of the other is not known (Fig. 5). Whether or not the flexibility in the composition of TFIIH is a more general aspect of its biology that may explain some aspects of the spatial and selective deregulation of nuclear receptor target genes in specific organs[63], or the lesion-specific DNA repair defect observed in XP, TTD, or CS patients[76], merits further investigations.

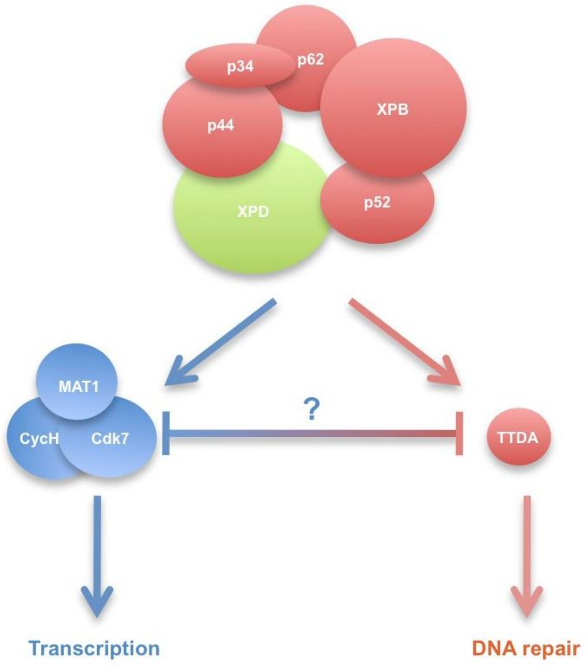

**FIGURE 5.** The CAK and TTDA modules engage the core TFIIH in different cellular pathways. TFIIH is composed of a core that associates with different modules to enable its engagement in different cellular processes. The presence of CAK in TFIIH engages the core in transcription[29], while the presence of TTDA engages the core in DNA repair[33]. The CAK and the TTDA modules coexist in the same TFIIH complex[33], but the negative influence of one module on the other is not known.

## ACKNOWLEDGMENTS

We are grateful Hans-Ulrik Kristensen for the critical reading of this manuscript. The work described here was supported by funds from the Ligue Contre le Cancer (Equipe Labellisée), from the French National Research Agency (ANR-08-GENOPAT-042), and from the Institut National du Cancer (INCA-2008-041). V.O is supported by the Association pour la Recherche contre le Cancer (ARC). FC would like to express his gratitude to David Mota, a former member of the lab, who left the group too prematurely (and in a rush!) to show his scientific and human talents. This paper is dedicated to the memory of Jonathan Gintz, a great colleague and friend who left us a year ago.
